# Femtosecond laser rapid fabrication of large-area rose-like micropatterns on freestanding flexible graphene films

**DOI:** 10.1038/srep17557

**Published:** 2015-11-30

**Authors:** Xuesong Shi, Xin Li, Lan Jiang, Liangti Qu, Yang Zhao, Peng Ran, Qingsong Wang, Qiang Cao, Tianbao Ma, Yongfeng Lu

**Affiliations:** 1 Laser Micro/Nano Fabrication Laboratory, School of Mechanical Engineering, Beijing Institute of Technology, Beijing 100081, PR China; 2 Key Laboratory of Cluster Science, Ministry of Education, School of Chemistry, Beijing Instituteof Technology, Beijing 100081, PR China; 3 State Key Laboratory of Tribology, Tsinghua University, Beijing 100084, PR China; 4 Department of Electrical Engineering, University of Nebraska-Lincoln, Lincoln, NE 68588-0511, USA

## Abstract

We developed a simple, scalable and high-throughput method for fabrication of large-area three-dimensional rose-like microflowers with controlled size, shape and density on graphene films by femtosecond laser micromachining. The novel biomimetic microflower that composed of numerous turnup graphene nanoflakes can be fabricated by only a single femtosecond laser pulse, which is efficient enough for large-area patterning. The graphene films were composed of layer-by-layer graphene nanosheets separated by nanogaps (~10–50 nm), and graphene monolayers with an interlayer spacing of ~0.37 nm constituted each of the graphene nanosheets. This unique hierarchical layering structure of graphene films provides great possibilities for generation of tensile stress during femtosecond laser ablation to roll up the nanoflakes, which contributes to the formation of microflowers. By a simple scanning technique, patterned surfaces with controllable densities of flower patterns were obtained, which can exhibit adhesive superhydrophobicity. More importantly, this technique enables fabrication of the large-area patterned surfaces at centimeter scales in a simple and efficient way. This study not only presents new insights of ultrafast laser processing of novel graphene-based materials but also shows great promise of designing new materials combined with ultrafast laser surface patterning for future applications in functional coatings, sensors, actuators and microfluidics.

Surface micro/nano patterning is a highly promising approach to control the optical, electrical, chemical, biological, mechanical and other properties of a solid surface^1^,^2^,^3^. The formation of micro/nano structures after surface patterning plays a key role in tuning the properties of materials and gives rise to diverse functions ranging from structural colors to tunable wetting properties^4^,^5^. Recently, as basic block of carbon nanomaterials, the single atomic carbon sheets of graphene become an attractive candidate in surface patterning due to its remarkable electrical, thermal, chemical, and mechanical properties^6^,^7^,^8^. Chemical vapor deposited (CVD) graphene and chemical exfoliated graphene oxides (GO) are commonly used as alternatives for micro/nano patterning due to their advantages in scalable and large-area preparation^9^. Existing studies of making patterns on CVD graphene and GO have been successful in tuning the dimension of graphene to obtain many in-plan graphene patterns, such as one-dimensional nanoribbons^8^,^10^ and two-dimensional nanodisks^11^ or square patterns^12^. For three dimensional (3D) hierarchical structures, two-step methods have been developed by deposition a layer of graphene on the surface of a pre-structured substrate^13^,^14^. However, this method is time-consuming owing to the multistep patterning and etching procedure in fabrication of the specific substrate. How to high-throughput fabrication of 3D hierarchical structures on graphene in a controlled manner still remains as open questions.

In general, scalable methods used to create surface patterns can be divided into two categories: bottom-up methods such as chemical synthesis, self-assembly and dip-pen nanolithography^15^,^16^, and top-down methods such as mask-less laser direct writing and focus ion beam etching^8^. For some functionalized films such as graphene and polypyrrole (PPy), subtractive patterning approaches are needed for increasing on-off ratio of graphene transistor or enhancing surface wettability^5^,^17^. While chemistry-based methods are versatile strategies for scalable bottom-up fabrication, mask-less laser direct writing is considered one of the most important research tool for top-down microstructuring of thin films^12^. Compared to other subtractive techniques, the advantage of laser direct writing is its simplicity, upwards scalability, high-throughput and low cost. Many lasers can be used as light sources in direct writing process, but not all of them are capable of making three-dimensional patterns on graphene films with high-throughput in large-scale. The femtosecond laser (fs) has been proved to have unique capabilities for scalable, high-throughput, controllable, and three-dimensional processing^18^,^19^,^20^. With ultrahigh peak powers (>10^13^ w/cm^2^) and ultrashort irradiation periods, fs laser fabrication presents unique advantages of nonlinear nonequilibrium processing^21^,^22^,^23^. The ultrafast localized nonlinear absorption leads to a spatial confinement of the radiation-induced material changes in a focal volume, which makes it possible to fabricate 3D geometrically complex structures with high-throughput^18^. On the other hand, the nonequilibrium effects enable photon-electron coupling to happen before the lattice changes. Hence, non-thermal mechanism can dominate the ablation process and thermal effects, such as heat-affected-zone and thermal damage can be significantly minimized^24^. Femtosecond lasers have been used to pattern surfaces with mico/nano structures for superhysdrophobic surfaces^25^,^26^. However, the formation surface micro/nano structures usually requires several to hundreds of fs pulses per unit area, which would decrease the efficiency of large-scale microstructuring.

In this study, large-area uniform 3D rose-like microstructures were fabricated using fs laser pulses. The 3D rose-like structure can be obtained by only one laser pulse, which offers great potential for the high-throughput large-scale manufacturing. The home-made freestanding graphene films, prepared from direct filtration of the aqueous reduced graphene oxide colloidal suspensions, were composed of both nanoscale graphene nanosheets separated by nanogaps and subnanoscle graphene monolayers with a large interlayer spacing of ~0.37 nm. The unique hierarchical structure offers great potential for the generation of tensile stress during fs laser ablation, which acted as a driven force for rolling up the graphene nanoflakes. Our approach shows potential application in fabrication of centimeter-scale 3D micro/nanostructures in a controllable and highly-efficient manner, which is distinct from earlier reports in that the graphene film itself served as an important precursor for the formation of graphene microflowers when it was processed with fs laser pulses.

## Results

**Figure 1:**
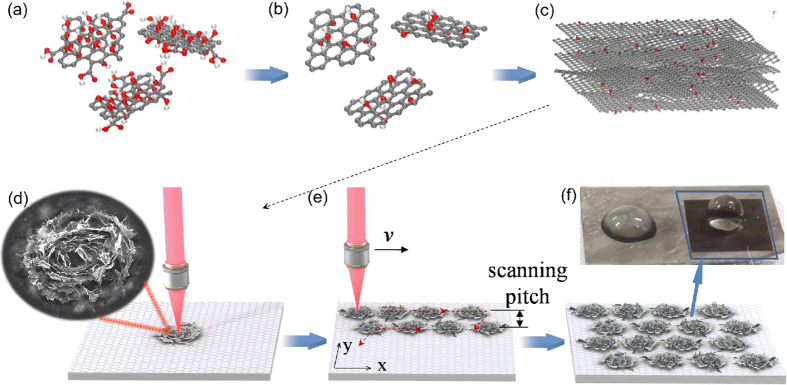
Schematics illustration of the procedures to produce the graphene film for surface patterning using femtosecond laser pulses. (**a**) Graphene oxide sheets. (**b**) Reduced graphene sheets. (**c**) Graphene film by filtration of reduced graphene sheets. (**d**) A graphene microflower produced with a single laser pulse at fluence of 1.1 J/cm^2^. (**e**) Large-area surface patterning by fs laser direct writing. (**f**) Large-area uniform flower patterns on the surface of a graphene film. Digital image of the pristine (left) and engineered surfaces (right, darker black) showing totally different surface wettability.

Figure 1 schematically illustrates the procedures to obtain large-area biomimetic micropatterns on graphene films. The graphene films were prepared by direct filtration of aqueous reduced graphene oxide colloidal suspensions through a filter membrane as detailed in our previous report^27^. After irradiation with a single laser pulse, a rose-like graphene microflower could be obtained in the ablated region, as shown in Fig. 1d. The laser beam was then scanned with coordinated adjustment of laser repetition rate, scanning speed (*v*), and scanning pitch (Fig. 1e). By moving the stage with respect to the laser beam, large-area arrays of biomimetic graphene microflowers with tunable densities were directly written (Fig. 1f). The patterned graphene area (3 × 3 mm^2^) shows superhydrophobility with a large water contact angle of 150°, whereas the original graphene film only exhibits hydrophilicity with a low water contact angle of 78° (Fig. 1f).

### Morphology and structure of graphene film

**Figure 2:**
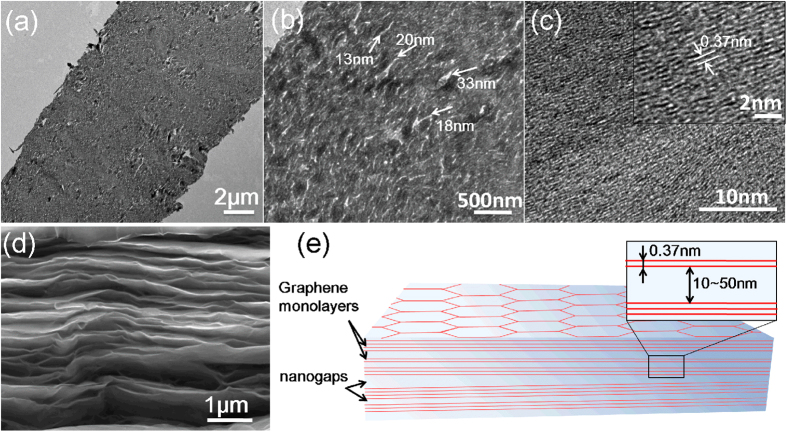
Morphology and structure of graphene film. (a) Low-, (**b**) middle-, and (**c**) high-resolution TEM cross-sectional images of the graphene film, which were obtained under a 300 keV electron beam. The arrows in (**b**) indicate the nanogaps of ~10–50 nm. The inset in (**c**) shows an image of the highest resolution. (**d**) SEM image of the fracture edge of the graphene film. (**e**) Schematic drawing of the structure of the graphene film.

The characteristic structure of the as-prepared graphene film was investigated by TEM and SEM, as shown in Fig. 2. Under the lowest magnification (Fig. 2a), it can be seen that the graphene film has a thickness of ~12 μm with front and back free surfaces. For a higher magnification (Fig. 2b), the nanogaps (indicated by the arrows) of ~10–50 nm among the graphene nanosheets can be clearly observed. With the highest magnification, well-stacked graphene monolayers can be observed in Fig. 2c, showing an interlayer spacing of ~0.37 nm, larger than that of typical graphite. Figure 2d shows SEM image of the fracture edge of the graphene film. According to the results of characterizations, the unique structure of the graphene film is schematically shown in Fig. 2e. The graphene film exhibits both nanoscale and sub-nanoscale structures. The nanoscale structure consists of graphene nanosheets, which are separated by randomly distributed nanogaps of ~10–50 nm. Each graphene nanosheet is composed of well-stacked graphene monolayers with an average interlayer spacing of ~0.37 nm. The aforementioned structure of graphene film presumably has significant effects on the subsequent fs laser ablation process (see details in the Discussion).

### Fabrication of graphene microflowers by fs laser pulses

**Figure 3:**
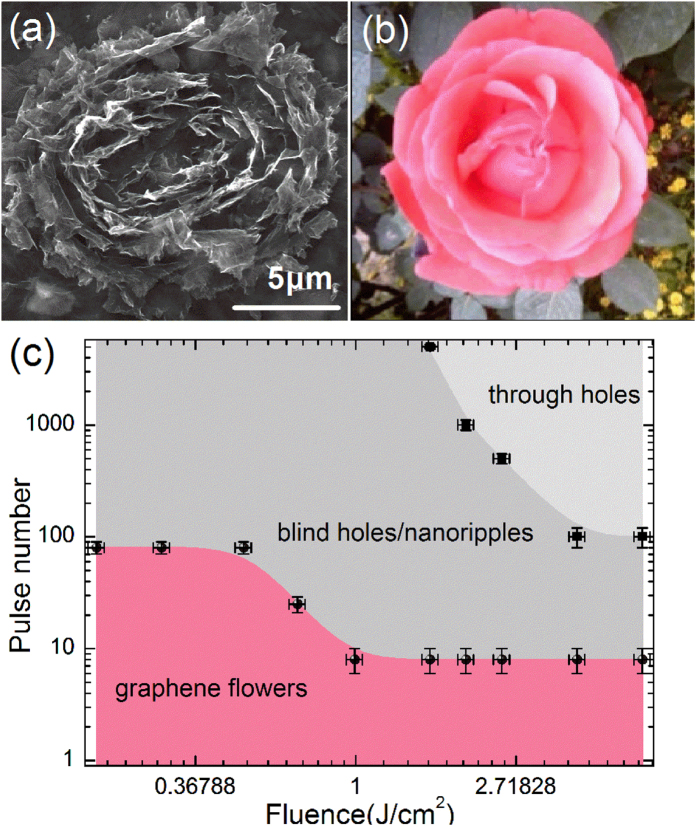
(**a**) SEM image of the biomimetic graphene microflower fabricated by a single fs laser pulse at fluence of 1.1 J/cm^2^. (**b**) Digital image of a Chinese rose. (**c**) The overview of processing parameters for the formation of various surface morphologies on graphene films. The pink shaded area indicates the laser exposure parameters for the formation of graphene microflowers.

After irradiation with a single laser pulse, a graphene microflower, exhibiting both micrometer-scale and nanometer-scale structures, could be fabricated at the laser fluence (F) of 1.1 J/cm^2^, as shown in Fig. 3a. Numerous turnup graphene nanoflakes were distributed around the central region of the ablated area. These graphene nanoflakes constitute the micro-sized graphene flower, which is similar to a blooming rose (Fig. 3b). The graphene microflowers could be fabricated at a relatively low pulse numbers (<100) around a wide range of laser fluences, indicated by the pink shaded area in Fig. 3c. In general, the three types of surface structures can be fabricated as shown in Fig. 3c. From the bottom to the top right, the surface morphology evolves from graphene microflowers to blind holes or nanoripples and to through holes (see the detailed structure morphologies in Supplementary Fig. S1). For comparison purpose, we also conducted laser ablation experiments on HOPG films composed of homogeneous graphene monolayers with an interlayer spacing of ~0.34 nm. The thickness of the HOPG films is ~ 13 μm, similar to that of the graphene films. In contrast to the graphene film, the microflowers were not obtained. After ablation with laser fluences ranging from 0.2 J/cm^2^ (slightly above the ablation threshold) to 4 J/cm^2^ (more than 20 times larger than the threshold), the ablated structures on HOPG films changed from smooth craters to nanoripples to through holes with the increase of the pulse number (see comparisons of the ablated structures between graphene film and HOPG film in Supplementary Fig. S1). Hence, the structures obtained on graphene films (microflowers) are totally different from that obtained on HOPG films (smooth craters) when irradiated with laser pulses at a relatively low pulse number.

**Figure 4:**
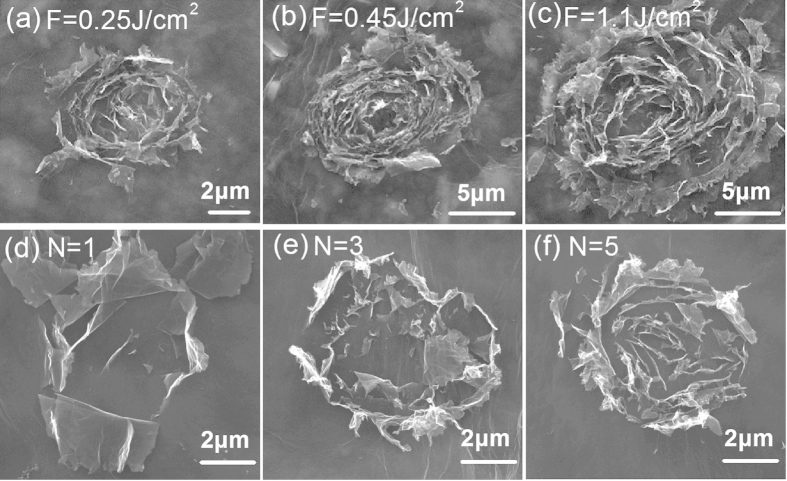
(**a–c**) Ablation size of graphene microflowers versus laser fluence after a single laser pulse ablation. (**d–f**) Evolution of the graphene microflowers with different pulse numbers at the same laser fluence of 0.2 J/cm^2^.

The size and shape of the graphene flower can be further controlled by tuning the laser fluence (F) and pulse number (N), respectively. Figure 4 a-c show that under a single pulse irradiation, the size of graphene microflowers increases with the increase of laser fluences, while the morphologies of the flowers approximately remain unchanged. Thus, we can precisely control the size of graphene flowers without changing their geometry by carefully adjusting the laser fluence. Figure 4d-f display the dependence of shape of graphene microflowers on pulse number at a relatively low laser fluence of F = 0.2 J/cm^2^. At N = 1, several intact and flat graphene flakes were peeled off from the center of the ablated region after a single pulse irradiation, leaving a relative clean and flat surface in the ablation crater (Fig. 4d). The fold of the underlying graphene sheet is clearly visible because the graphene sheets are very thin. As the pulse number increased to N = 3, a new array of graphene nanoflacks were exfoliated from the “new surface” of the graphene film that generated by the previous pulse (Fig. 4e). In this case, most of the ablation area was dominated by the roll-up graphene nanoflacks except the central region. At the pulse number of N = 5, much more graphene nanoflakes were rolled up from center to periphery of the crater, which can dominate the whole area of the ablated region (Fig. 4f). Hence, the “growth” and shape of the microflowers can be controlled by tuning the number of fs laser pulses.

### Large-area uniform flower patterns with tunable densities and wetting properties

**Figure 5:**
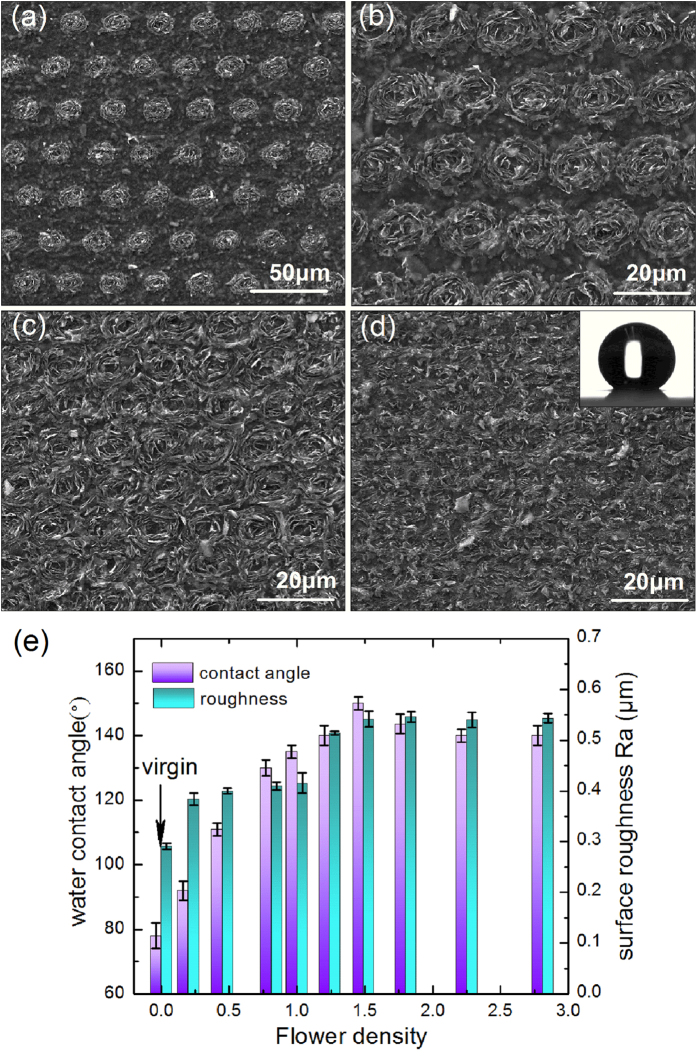
(**a–d**) Fs laser large-area fabrication of graphene films with different densities of flower patterns. The inset image in (**d**) shows the water droplet on the patterned graphene film surface with a contact angle of ~150°. (**e**) Dependence of the contact angle and surface roughness on the density of the graphene microflowers, D, from 0 to 2.81.

Large-area micropatterning of graphene films with controllable densities of flower patterns was realized by a simple scanning technique with coordinated adjustments of laser repetition rate, scanning speed (*v*), and scanning pitch. The details of the flower density (D) calculation, and the relation between scanning parameters and flower densities were described in Supplementary Table S1. Figure 5a–d show the SEM images of graphene films with different densities of flower patterns fabricated by fs laser direct writing at a fixed fluence of 1.1 J/cm^2^. It is shown that the graphene microflowers within the scanning area have almost the same geometrical feature, indicating the excellent repeatability. The density of graphene microflowers increases gradually from Fig. 5a–d, among which the surface with microflowers overlapped with each other in Fig. 5d shows the highest contact angle (CA) of ~150°. The static water contact angles of the micropatterned graphene films were measured to investigate the dependence of wettability on the density of flower patterns, as shown in Fig. 5e. The untreated graphene film exhibits hydrophilicity with CA of ~78°, while the CA of patterned surface increases dramatically to ~150°, reaching the superhydrophobic level. As the flower density can be tuned continuously, the contact angles between 78° and 150° can also be controlled.

**Figure 6:**
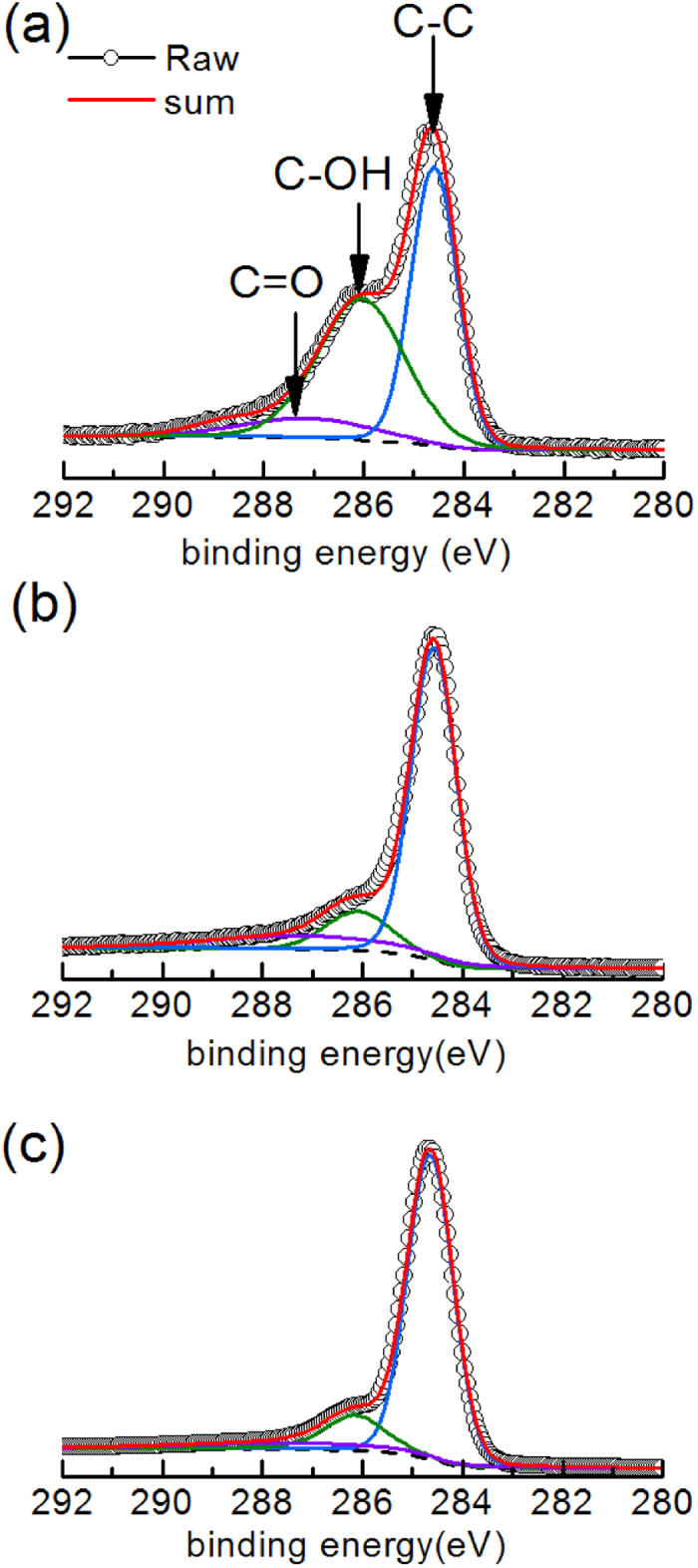
C1s XPS spectra of the pristine and patterned graphene films. (**a**) Pristine graphene film without surface patterning. (**b,c**) Patterned graphene films with increasing flower densities. The densities in (**b,c**) are equal to those in Fig. 5c and Fig. 5d, respectively.

To further substantiate the effects of laser patterning on the wetting properties of graphene films, XPS and AFM were used to investigate the chemical composition and the surface geometry, respectively. The oxygen content of the pristine surface and patterned graphene films with flower densities equal to that in Fig. 5c and d were measured by C1s XPS. As shown in Fig. 6, the binding energy of the C-C is assigned at 284.6 eV and chemical shifts of + 1.5 and + 2.5 are assigned for the C-OH and C = O functional groups, respectively^28^. The XPS results show that the oxygen content of the pristine graphene film is 29.03%, while the patterned surfaces with microflowers just next to each other (Fig. 5c) and partially overlapped (Fig. 5d) are 12.39% and 6.76%, respectively. The XPS results indicate that the oxygen groups have been successfully reduced by fs laser ablation and the oxygen content decreases with the increase of the flower density. Hence, a further removal of hydrophilic oxygen groups by fs laser ablation may play an important role in the enhanced hydrophobicity of the patterned graphene films. Besides, the surface roughness (Ra) of graphene films with different flower densities was also investigated by AFM as shown in Fig. 5e. Generally, the CA changes according to the surface roughness. Because of the turnup graphene flakes, the edges of the exfoliated graphene flakes were exposed to the surface. On the surfaces with plenty of graphene edges, a mix of solid/liquid and liquid/air interfaces can be created compared to the solid/liquid interface on the plain samples, resulting in an increasing water contact angle^26^. However, further increase of the flower density (i.e. D > 1.49) leads to a slight decrease of the contact angle. According to the scanning technique, a larger flower density means more laser pulses applied on unit area. The additional pulses applied on the preexisting microflowers can result in an increase of the density of graphene flakes but a decrease in volume of cavities between the turnup nanoflakes, which may be responsible for the slight decrease in the contact angle at a larger flower density.

In addition, we found that the obtained superhydrophobic surfaces also exhibit high adhesion to water. The critical volume of water droplet hanging upside down was ~15 μL, which indicates that the highest retention force is about 150 μN. A 15 μL water droplet that suspended on an upside down graphene film is shown in Supplementary Fig. S2. Because the graphene microflowers consist of large numbers of turnup graphene flakes, we infer that 1) a high van der Waals’ force between the edges of graphene flakes and the liquid interface could be generated;^29^ 2) sealed air could be trapped within the nanogaps between the turnup graphene flakes to generate negative pressure when the volume of the air changes^30^. Both of the aforementioned aspects might be responsible for the high adhesion force.

## Discussion

It is worth noting that the microflowers can be obtained on graphene films that have unique hierarchical layering structures, while they cannot be fabricated on HOPG films with a uniform interlayer spacing. On the other hand, the microflowers consist of graphene nanoflakes that were exfoliated from the surface by fs laser pulse, which are geometrically similar to the fragmentation of thin spalls induced by tensile stress^31^. Considering the above-mentioned aspects, we proposed that the unique hierarchical structure of the graphene film provides significant possibilities in the generation of tensile stress during femtosecond laser ablation to roll up the graphene nanoflakes. During fs laser ablation, due to the high peak power (>10^13^ W/cm^2^), high-density (>10^21^ cm^−3^) electrons^22^ can be excited on the material surface. The pulse energy absorption by the electrons leads to a rapid increase in the electronic temperature, creating an electron-ion non-equilibrium state. The absorbed energy was then transferred to lattice via electron-phonon coupling, which leads to a strong temperature gradient within a thin layer below the front surface. Because the duration of a femtosecond laser pulse is much shorter than the time of mechanical equilibration of the absorbing volume, the stress can be confined temporarily result from the fast energy deposition in the absorption region^32^. Hence, the strongly overheated layer is in a highly compressed non-equilibrium state, which will lead to phase transitions and an ejection of the surface materials towards the laser source at high speed rates^31^. The laser-induced material ejection from graphene film was confirmed by pump-probe shadowgraphy, as shown in Supplementary Fig. S3. Shadowgraphs show the outward expansion of the ejected material, which is enwrapped by the shock wave front. It has been reported that the fast ejection of the material can result in shock waves propagating both outside and inside the target^33^. The shock wave propagating inside the material is called as stress wave, which can be formed under conditions of partial inertial stress confinement when the material is rapidly heated by ultrashort laser pulses^32^. Initially, the stress wave yields a compression and propagates in-depth within the material. When the compressive wave is reflected from a free boundary, it becomes tensile^34^,^35^. The significant magnitude of the reflected tensile stress may cause nonthermal ablation or the spallation within the material^34^,^36^. In our experiments, because the nanogaps (~10–50 nm) within the graphene film are much larger than the equilibrium bond distance, the van der Waals’ force between graphene nanosheets can be neglected where nanogaps exist. The corresponding partial graphene sheet can be treated as a “freestanding” thin fragment approximately. Hence, tensile stress waves could be generated after reflection by these “free surfaces”. The tensile stress may act as a driven force to roll up the graphene nanoflakes for the formation of graphene microflowers. The laser-induced lattice modification in graphene film was investigated by Raman microscopy (as details in Supplementary Fig. S4). On the other hand, the graphene films were prepared by assembly of exfoliated reduced graphene oxide sheets through a filtration method, resulting in many defect sites such as edges of nanosheets within the film. When the maximum of tensile stress reaches the fracture threshold, cracks can be formed preferentially along the weakest mechanical parts, i.e. edges between adjacent graphene flakes. The formation of cracks enables the occurrence of new “free surfaces”, where the compression stress can be released. If the maximum of the initial compression wave is high enough, a multiple spallation process is possible during the action of a single pulse^37^. In addition, the two-dimensional graphene nanosheets are connected by relatively weak intermolecular interactions including van der Waals and electrostatic bonds^38^, which can prevent the necessity of breaking in-plane covalent bonds to obtain a peeled nanoflake from the film surface. Compared to the graphene films, the high-quality (defect-less) HOPG film consists of homogeneous graphene monolayers with an interlayer spacing of ~0.34 nm. Fs laser ablation of HOPG films cannot lead to the formation of microflowers, but only smooth craters (see Supplementary Fig. S1). The experimental results confirm that the unique edge defects and nanogpas within the graphene film are mainly responsible for the formation of unique microflowers.

In summary, a simple and efficient method for large-area fabrication of 3D biomimetic microflowers on the graphene films with hierarchical layering structure have been developed using femtosecond laser pulses. The unique structure of the graphene films offers great potential for the generation of tensile stress during femtosecond laser ablation to roll up the graphene nanoflakes for the formation of a complex microflower by only a single laser pulse. Moreover, the size and shape of the microflowers can be precisely controlled by tuning the laser fluence and pulse number, respectively. After large-scale fabrication, the surface of graphene film was covered with uniform arrays of flower patterns with controllable density, exhibiting superhydrophobility and a high retention force to water. The fabricated biomimetic microflowers on the freestanding and flexible graphene films can find broad applications in bionics, microfluidics, sensors, micro/nano-droplets manipulation, single droplet analysis, Janus interface materials for actuators, and in fabrication of lab on a chip that need to retain tiny quantities of liquid without leaking.

## Methods

### Preparation of graphene films and HOPG films

The graphene films were prepared by direct filtration of aqueous reduced graphene oxide colloidal suspensions through a filter membrane as detailed in our previous report^22^. The dried graphene films were peeled off from the filter. The freestanding graphene film has a thickness of ~12 μm (see Supplementary Fig. S5) and an interlayer spacing of ~0.37 nm, larger than that of highly oriented pyrolytic graphite (HOPG) (~0.34 nm, measured by XRD, see Supplementary Fig. S6). A small amount of oxygen could be detected in the graphene films (by EDS, see Supplementary Fig. S7). The D peak in Raman spectra indicates structural disorder of the films (see Supplementary Fig. S4). The measured conductivity of the graphene film is ~1900 s/m. Scotch tapes were used to achieve the HOPG films with thickness of ~13 μm (see Supplementary Fig. S8).

### Femtosecond laser micropatterning

The setup of the fabrication system was described in Supplementary Fig. S9. A 3.5 W 50 fs Ti: sapphire laser (Spectra Physics, Inc.) was used to generate linearly polarized laser pulses at a central wavelength of 800 nm. The graphene film was mounted on a six-axis stage (M-840.5DG, PI, Inc.) with a maximum speed of 2 mm/s. The linear polarized laser beam was incident normal to the sample surface by a 5 × microscope objective (N.A. = 0.15, Olympus), corresponding to a spot size diameter of ~8 μm. The laser pulse energies were measured just before the objective lens. One pulse per site was realized by scaling down the laser repetition rate and adjusting the laser scanning speed.

### Pump-probe measurement

The pump-probe experiments were conducted with the same fs laser. A laser beam was divided into a pump and a probe beam by a beam splitter. The pump beam was focused onto the target surface by a 5 × objective lens. The probe beam was frequency doubled to 400 nm by a beta barium borate crystal. Using a CCD camera, the shadowgraphs of the early plasma were detected.

### Characterization

The surface morphology was investigated by a scanning electron microscope (SEM, FEI Quanta 200 FEG, Holand FEI Company). The average interlayer spacing was evaluated by a parallel beam Bruker D8 Advance X-ray diffractometer (XRD). The electrical conductivity was measured using a four-probe conductivity test meter (KDY-1, Kunde Technology, Co., Ltd.) at room temperature. Raman spectra were obtained using a Renishaw InVia Reflex spectrometer with a 532 nm light source at a low power. The transmission electron microscopy (TEM) was performed using an FEI Tecnai G2 F30 transmission electron microscope. Roughness measurements were carried out using an atomic force microscope (AFM, Veeco Dimension V). The water contact angles were measured using a K12 tensiometer (Kruss Company, Germany). X-ray photoelectron spectroscopy (XPS) was carried out using a PHI Quantera X-ray photoelectron spectrometer.

## Additional Information

**How to cite this article**: Shi, X. *et al.* Femtosecond laser rapid fabrication of large-area rose-like micropatterns on freestanding flexible graphene films. *Sci. Rep.* **5**, 17557; doi: 10.1038/srep17557 (2015).

## Supplementary Material

Supplementary Information
