# Supplementary material for: Femtosecond laser rapid fabrication of large-area rose-like micropatterns on freestanding flexible graphene films
Source: Sci Rep. 2015 Nov 30;5:17557. doi: 10.1038/srep17557 (PMC4663466; doi:10.1038/srep17557)
Supplement: Supplementary Information [file srep17557-s1.pdf]

Femtosecond laser rapid fabrication of large-area rose-like micropatterns on freestanding flexible graphene films

Xuesong Shi,[1] Xin Li,[1] Lan Jiang,[1,*] Liangti Qu,[2] Yang Zhao,[2] Peng Ran,[1] Qingsong Wang,[1] Qiang Cao,[1] Tianbao Ma,[3] and Yongfeng Lu[4]

[1]Laser Micro/Nano Fabrication Laboratory, School of Mechanical Engineering, Beijing Institute of Technology, Beijing 100081, PR China, [2]Key Laboratory of Cluster Science, Ministry of Education, School of Chemistry, Beijing Instituteof Technology, Beijing 100081, PR China, [3]State Key Laboratory of Tribology, Tsinghua University, Beijing 100084, PR China, [4]Department of Electrical Engineering, University of Nebraska-Lincoln, Lincoln, NE 68588-0511, USA

*jianglan@bit.edu.cn

Supplementary Materials

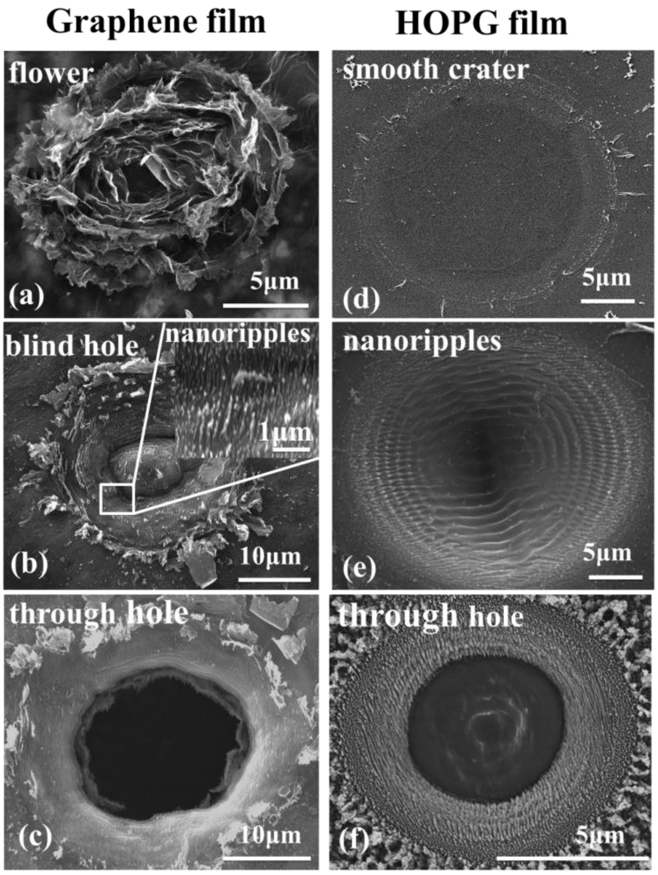

Figure S1 | Comparisons of typical surface morphologies obtained on graphene films (a)-(c) and HOPG films (d)-(f) by fs laser irradiation under different fluences, F, and pulse numbers, N. (a) Graphene flower obtained at N = 1, F = 1.1 J/cm^2; (b) Nanoripples with ~80 nm periods obtained at N = 1000, F = 1.6 J/cm^2 ; (c) Through hole obtained at N = 5000, F = 4 J/cm^2; (d) Smooth crater obtained at N = 1, F = 2 J/cm^2; (e) Nanoripples obtained at N = 100, F = 2

J/cm^2; (f) Through hole obtained at N = 1000, F = 1.1 J/cm^2

Calculation of density of microflowers

The density (D) of flower patterns was defined as the ratio of the pulse number (N) applied on a 1×1 mm^2 area to a reference value (Nr). The reference value, Nr, was defined as the applied pulse number on a 1×1 mm^2 area, in which graphene microflowers are "just" next to each other, i.e., the unit size is 12 μm × 15 μm. The pitch in Y direction (12 μm) is smaller than the step in X direction (15 μm) because of the return error of the stage. Hence, the defined reference value is Nr = 5561. The densities of flower pattern calculated for different parameter combinations are listed in Tab. S8, where D = 0 represents the pristine graphene film, D = 1 refers to the above mentioned reference case, and D > 1 means the graphene microflowers were overlapped with each other. Because the graphene microflower can be fabricated with a single pulse, one pulse per site was realized by scaling down the laser repetition rate due to the limited speed of stage (2 mm/s). Future use of fast scanning techniques, such as galvanometric scanners, will make large-scale fabrication much more efficient.

Table S1 | Densities of flower patterns calculated for different combinations of scanning speed, repetition rate and scanning pitch.

Density (D)	0.20	0.45	0.81	1	1.24	1.49	1.80	2.25	2.81
Scanning speed (μm/s)	1500	2000	1500	1500	1200	1200	2000	2000	2000
Repetition rate (Hz)	50	100	100	100	100	100	200	200	250
Scanning interval (μm)	30	20	15	12	12	10	10	8	8

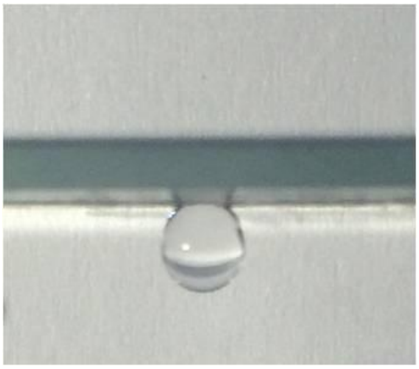

Figure S2 | Photo of a 15 μL water droplet suspended on the upside down surface of a patterned graphene film. The graphene film was fixed on a slide.

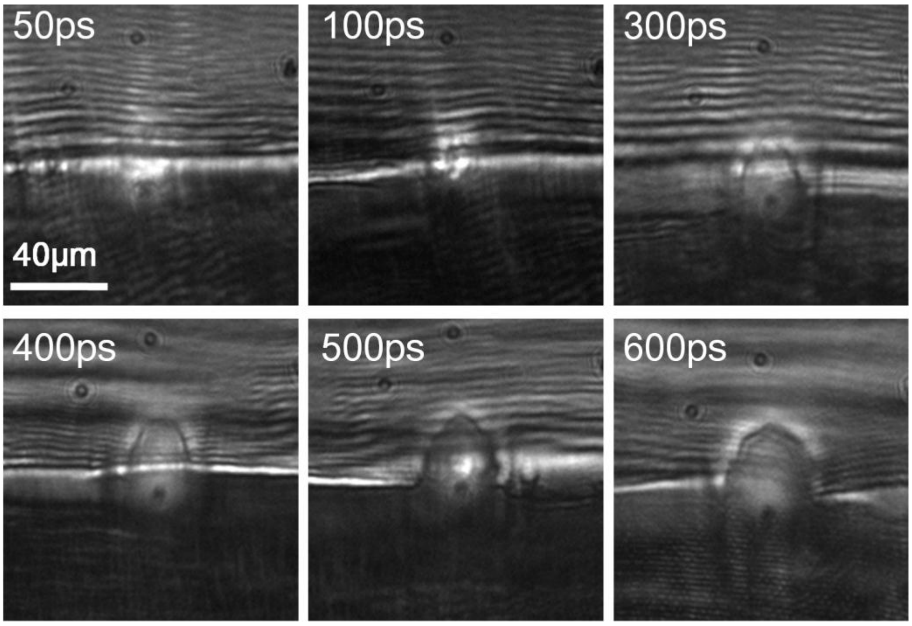

Figure S3 | Time-resolved images of plasma generated by fs laser ablation of graphene films at different delay times. Due to edge diffraction of the probe beam, there are some fringes parallel to the surface of graphene films. Based on the pump-probe results, the average plasma expansion velocity within 600 ps was ~5×10^4 m/s.

Laser-induced lattice modification in graphene film

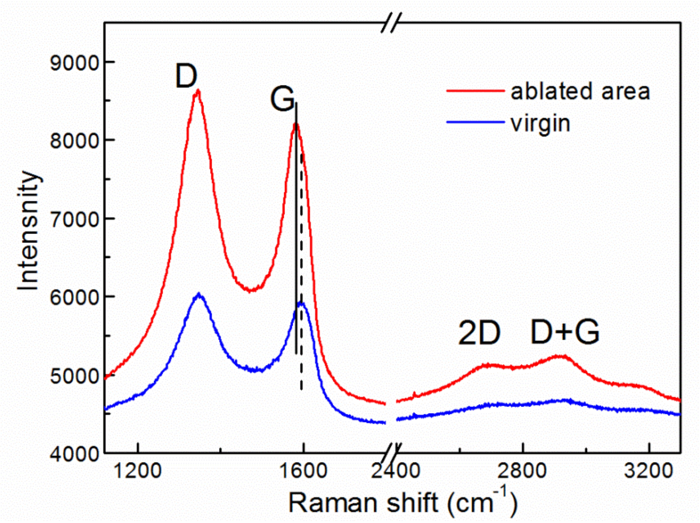

Figure S4 | Raman spectrum of the graphene films before and after ablation.

Compared to the pristine region, Raman spectra of the ablated area consist of G (1581 cm^{-1}), D (1345 cm^{-1}) lines, and some second-order Raman features, including 2D band at 2690 cm^{-1} and D + G combination band at 2930 cm^{-1}, as shown in Fig. S7. Firstly, the peak at the G band (1581 cm^{-1}) obtained after ablation is shifted to lower frequency (red shift) by 11 cm^{-1} compared to that before ablation, which is nearly consistent with that for graphite (1580 cm^{-1}). This can be attributed to the recovery of the hexagonal network of carbon atoms with defects by the reduction of oxygen. Secondly, the increase in D band represents an introduction of defects in the lattice after laser ablation. The ID/IG ratio of the ablated material (1.05) is larger than that of the pristine material (1.02), indicating that the ablation process altered the crystal structure of graphene film with more structural defects. Thirdly, a slight increase in the intensity of 2D (2690 cm^{-1}) and the D + G combination band (2930 cm^{-1}) can be observed for the ablated region, which may be attributed to that the sample contains highly disordered and randomly arranged graphene sheets after laser ablation.

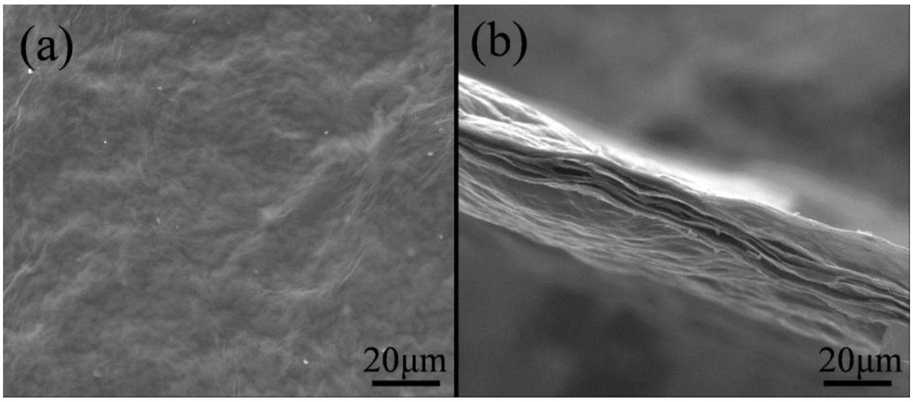

Figure S5| SEM images of top (a) and cross-section (b) view of the as-prepared graphene film.

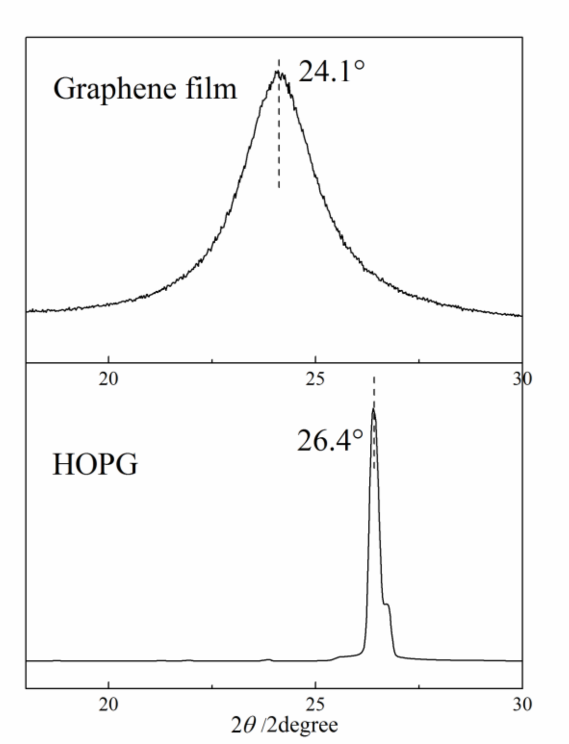

Figure S6 | XRD spectrums of the as-prepared graphene film and HOPG film.

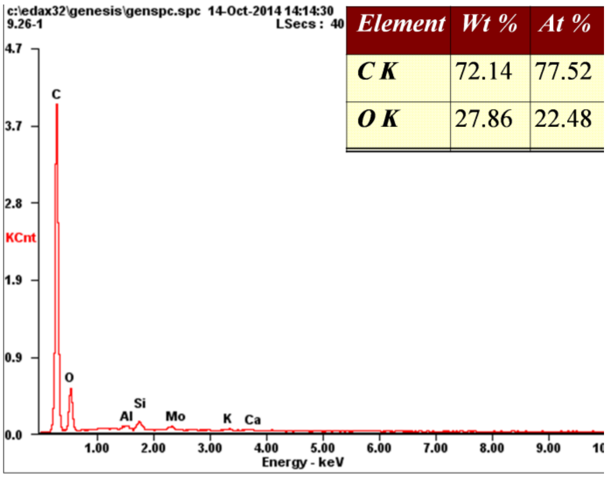

Figure S7 | EDS spectrum of the as-prepared graphene film.

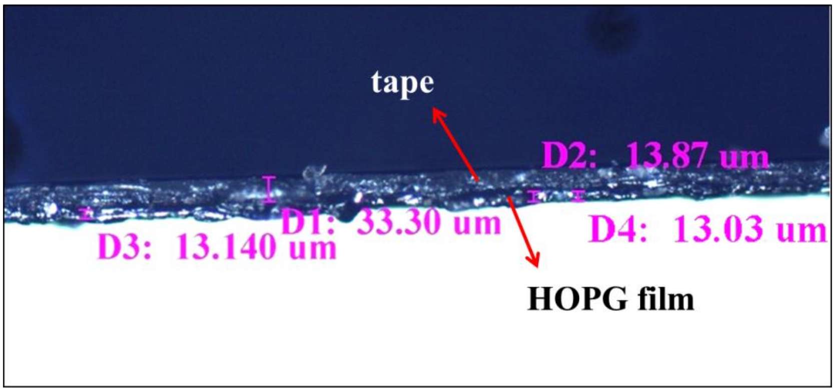

Figure S8 | Optical microscope image of HOPG film sticking on the tape.

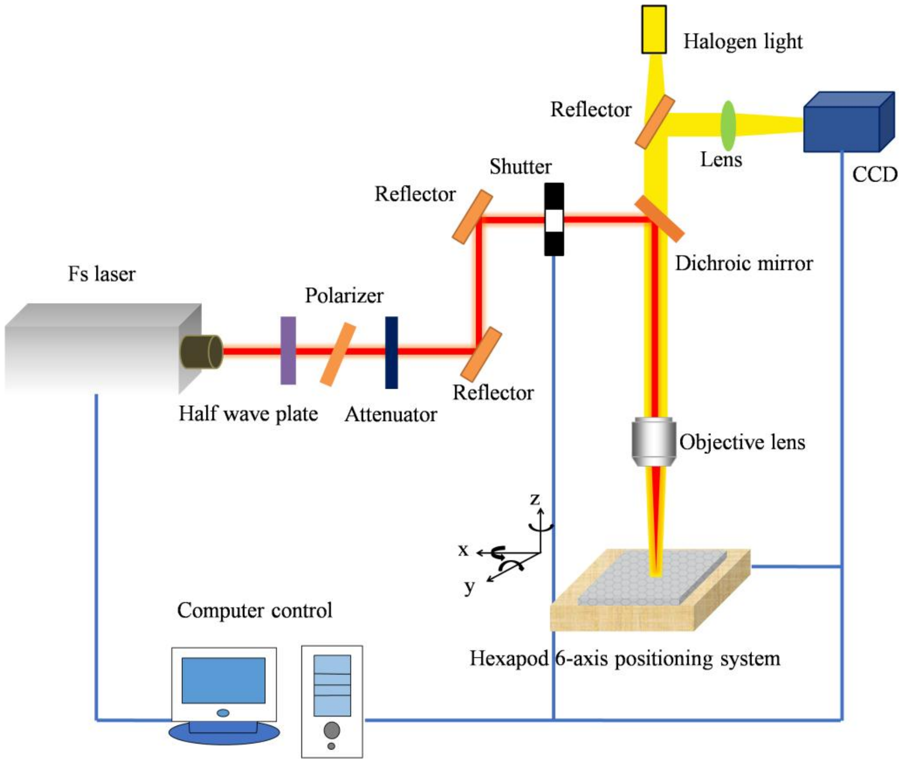

Figure S9 | Experimental setup for micropatterning of graphene films with femtosecond laser direct writing.
